# MUC1 in Colorectal Carcinoma: Association With Prognosis and Putative Anoikis‐Resistant Structures

**DOI:** 10.1111/apm.70105

**Published:** 2025-12-02

**Authors:** Taneli T. Mattila, Madhura Patankar, Juha P. Väyrynen, Kai Klintrup, Jyrki Mäkelä, Anne Tuomisto, Pentti Nieminen, Markus J. Mäkinen, Tuomo J. Karttunen

**Affiliations:** ^1^ Department of Pathology, Research Unit of Translational Medicine University of Oulu Oulu Finland; ^2^ Department of Pathology, Oulu University Hospital and Medical Research Center Oulu Oulu Finland; ^3^ Department of Molecular and Cellular Biology University of California Davis Davis California USA; ^4^ Department of Surgery, Oulu University Hospital and Medical Research Center Oulu Oulu Finland; ^5^ Department of Surgery, Research Unit of Surgery, Anesthesia and Intensive Care University of Oulu Oulu Finland; ^6^ Medical Informatics and Data Analysis Research Group University of Oulu Oulu Finland

**Keywords:** anoikis, colorectal carcinoma, cribriform, micropapillary, MUC1, solid

## Abstract

Mucin 1 (MUC1) is overexpressed in colorectal cancer (CRC), yet its prognostic value is controversial. In vitro, MUC1 promotes anoikis resistance. Putative anoikis‐resistant (AR) structures are clusters of carcinoma cells surviving without matrix contact, and are associated with adverse prognosis. We explored the prognostic role of MUC1 in CRC and its association with putative AR structures. We studied 118 patients with CRC, including 52 with nodal metastases. Immunohistochemical MUC1 expression was analyzed in primary tumors and nodal metastases by estimating the proportions of MUC1‐positive carcinoma cells within different subpopulations. In primary tumors, MUC1 expression was increased in most putative AR structure types, and abundant AR structures were associated with elevated MUC1. MUC1 in primary tumors was not prognostic. In nodal metastases, MUC1 levels correlated with those in primary tumors but were lower. High MUC1 levels in both primary tumors and metastases were associated with worse prognosis as compared with expression decreasing in nodal metastases. High MUC1 expression in nodal metastases was associated with synchronous distant metastasis and adverse prognosis. In conclusion, primary tumor MUC1 is not prognostic. In nodal metastases, high MUC1 associates with distant metastasis and poor outcomes. Putative AR structures show high MUC1 expression supporting its role in anoikis resistance.

## 1 Introduction

Colorectal carcinoma (CRC) accounts for one in 10 cancer‐related deaths worldwide [1]. New therapeutic strategies and reliable prognostic and predictive factors are needed, especially in locally advanced CRC [2]. Despite the heterogeneity of advanced‐stage CRC in terms of prognosis [3], relatively few studies have addressed the prognostic or predictive features in nodal or distant metastases [4, 5, 6, 7]. While adjuvant chemotherapy is often considered, identifying markers to gauge the risk of concurrent or future development of distant metastases is important for optimal patient management.

Mucin 1 (MUC1, epithelial membrane antigen, EMA) is a transmembrane glycoprotein [8] expressed by normal columnar epithelial cells, with increased expression in malignant cells including CRC [9, 10]. In vitro, overexpression of MUC1 can enhance cell proliferation, invasion, chemoresistance, and apoptosis inhibition [11, 12, 13, 14, 15, 16], with the latter considered its most consistent effect [11]. Although high MUC1 expression in primary CRC has been linked to advanced disease and poor prognosis [17, 18], many earlier immunohistochemical studies [17, 18, 19, 20] relied on biotin‐based detection methods prone to false positives in many tissues including colon [21]. More recent polymer‐based detection studies have shown either negative, or limited prognostic significance of MUC1 only in mismatch repair–proficient cancers [22, 23]. Hence, the overall prognostic value of MUC1 in CRC remains uncertain.

Anoikis is programmed cell death triggered by loss of extracellular matrix (ECM) attachment [24]. Resistance to anoikis allows cancer cells to survive without ECM contact, enabling metastasis [25, 26, 27] and contributing to the selection of competent tumor cell subclones to form metastatic lesions [28, 29]. MUC1 has been suggested to play an important role in AR. The O‐glycosylated form of MUC1 is able to prevent activation of surface molecules that induce anoikis [14]. Additionally, the subcellular distribution of MUC1 is related to glycosylation status, and loss of normal apical polarity in malignant cells, reinforces its potential importance in cancer progression [13, 30, 31].

We recently introduced a method to identify putative AR subpopulations in CRC using standard histopathology [32]. These subpopulations include cells in micropapillary, cribriform, or solid structures, all of which lack basement membrane contact. Their abundance correlates with worse prognosis [32] and they are more prevalent in nodal metastases [28], possibly reflecting a survival advantage for cells capable of forming such structures. Consistent with this concept, in vitro 3D models have shown that anoikis is crucial for hollow lumen formation in mammary acini, while oncogene activation can lead to inhibition of anoikis and formation of cribriform or solid structures [33]. Similarly, in Caco‐2 intestinal cancer cells natively forming cysts in 3D cultures, insertion of BRAF oncogene induces anoikis resistance and formation of micropapillary‐like and solid structures [34], supporting the biological relevance of such growth patterns in CRC.

Although upregulation of MUC1 has been implicated in anoikis resistance, direct evidence for such an effect in human CRC is lacking. Furthermore, the prognostic role of MUC1 remains debated. In this study, we analyzed MUC1 immunohistochemical expression in CRC primary tumors and lymph node metastases to clarify a role in anoikis inhibition. We analyzed whether putative AR subpopulations display different MUC1 patterns as compared with other carcinoma cells and whether there is any association with cell proliferation and apoptosis rates. We also evaluated any prognostic impact of overall MUC1 expression and that in putative AR subpopulations. To see whether there is any evidence for a selection of any expression pattern along with the formation of metastases we compared the expression patterns of MUC1 in nodal metastases and primary tumors. Finally, we were interested to see whether changed expression of MUC1 in lymph node metastases associated with distant spread of carcinoma or worse outcomes.

## 2 Materials and Methods

### 2.1 Patients

The present study is based on the same prospective series of 149 patients diagnosed with CRC who underwent surgery at Oulu University Hospital between 2006 and 2010 (Table 1), as previously described in our prior publications [28, 32, 35]. Of the cases, 31 were excluded due to insufficient sample material, resulting in 118 cases in total. Of these, 52 had nodal metastasis, 46 cases having sufficient sample material including corresponding primary tumors. Preoperative radiation therapy was given to 22 cases and they were included in the study. Clinical and follow‐up data were collected from the patients' clinical records and Statistics Finland. For the assessment of cancer‐specific survival (CSS), we measured the time from the operation to cancer‐related death. This research project received approval from the Ethics Committee of Oulu University Hospital (58/2005, 184/2009).

**TABLE 1:** Clinical and pathological features of colorectal carcinoma cases (*n* = 118) analyzed.

Variables	Category	*n* (%)
Total AR	High (> 6.86 structures/mm^2^)	21 (17.8)
	Low	97 (82.2)
Age, mean (SD)	67.0 (11.0)	
	≤ 65	44 (37.3)
	> 65	74 (62.7)
Sex	Male	62 (52.5)
	Female	56 (47.5)
Localization	Proximal colon	37 (31.4)
	Distal colon	25 (21.2)
	Rectum	56 (47.5)
Preoperative therapy (rectal only)	Yes	22 (39.3)
	No	34 (60.7)
WHO Grade	Well differentiated	13 (11.1)
	Moderately differentiated	89 (76.1)
	Poorly differentiated	15 (12.8)
TNM Stage	I	18 (15.3)
	II	44 (37.3)
	III	39 (33.1)
	IV	16 (13.6)
Distant metastasis	Yes	16 (13.7)
	No	101 (86.3)
Lymph node metastasis	Yes	52 (44.4)
	No	65 (55.6)
Number of metastases, mean (SD)	6.21 (9.97)	
Extranodal extension	Yes	26 (59.1)
	No	18 (40.9)
Lymphatic invasion	Yes	53 (45.3)
	No	64 (54.7)
Blood vessel invasion	Yes	24 (20.5)
	No	93 (79.5)
Infiltrating border	Yes	32 (27.4)
	No	85 (72.6)
Cancer type	Conventional adenocarcinoma	91 (77.1)
	Serrated adenocarcinoma	27 (22.9)
Mismatch repair (MMR)	Proficient	110 (94.0)
	Deficient	7 (6.0)
BRAF mutation	Yes	9 (7.8)
	No	107 (92.2)
KRAS mutation	Yes	29 (27.1)
	No	78 (72.9)

### 2.2 Histology and Immunohistochemistry

#### 2.2.1 Histology

Histological evaluation of the primary tumors, including grading and staging, was conducted using whole slide sections stained with hematoxylin and eosin (H&E) [35]. To assess putative anoikis‐resistant (AR) structures in primary tumors, we employed H&E‐stained tissue microarrays (TMAs) [35], and for lymph node metastases, we used whole slide images [28]. The sections were digitally scanned using 20× objective magnification for analysis with the Leica‐Aperio AT2 system from Leica Biosystems. The outcomes of these investigations on putative AR subpopulations in H&E‐stained sections have been previously published [28, 32].

For the assessment of MUC1 expression, formalin‐fixed, paraffin‐embedded sections of primary tumor TMAs and nodal metastases were immunohistochemically stained with the Bond RX stainer (Leica Biosystems, Nussloch, Germany). Sections were treated with Leica Bond Epitope Retrieval Solution 2. Primary antibody (monoclonal mouse, Anti‐Human Epithelial Membrane Antigen, clone E29, code M 0613, dilution 1:500; Dako, Copenhagen, Denmark) was incubated for 30 min at room temperature. MUC1 E29 antibody recognizes the glycosylated antigen and has been clinically validated [36]. For the detection of bound antibodies, we used Leica Bond Polymer Refine Detection DS9800 (Leica Biosystems), a polymer‐based, biotin‐free detection system. The immunohistochemistry slides were digitized using 40× objective magnification with the Leica‐Aperio AT2 system (Leica Biosystems). For cases with more than one TMA core analyzed or more than one lymph node metastasis, the mean was calculated to represent the primary tumor or metastases of the case.

### 2.3 Detection of Putative Anoikis‐Resistant Structures

As previously reported [28, 32], putative AR structures were quantified by a specialist in anatomical pathology (T.T.M.) who was blind to any clinical data and scoring results of corresponding primary tumors and nodal metastases. We employed previously established criteria for identifying three types of putative AR structures [32]:

- MIPs (Micropapillary structures):

These are cells forming stacks at the luminal side of glandular structures, with a minimum pile thickness of two cells and a lateral extent of at least two cells.

ii Cribriform structures:

These are groups of cells at least four cells thick, with scattered empty spaces present among the cells.

iii Solid structures:

These consist of groups of cells at least four cells thick, forming solid sheets.

All putative AR structures were visually identified in each TMA core and metastasis, and their areal density expressed as number of structures/mm^2^ of tumor tissue [28]. Additionally, the sum of the densities of the three AR structure types was calculated and termed Total AR density.

### 2.4 Scoring of MUC1 Expression

**FIGURE 1:**
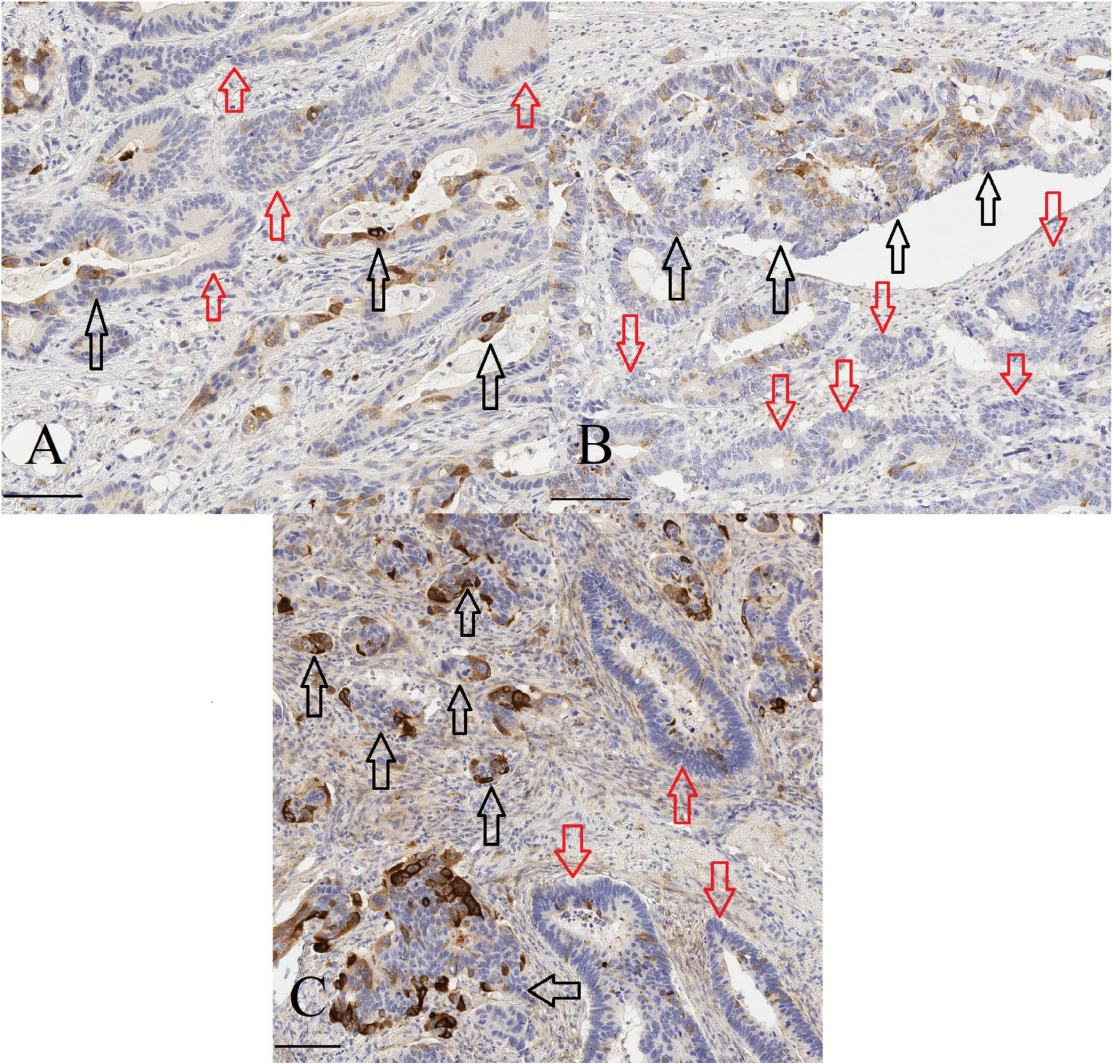
Examples of MUC1 (E29) immunohistochemistry in colorectal carcinoma primary tumors showing various staining patterns in different subpopulations of the carcinoma cells. Black arrows mark putative anoikis‐resistant subpopulations including micropapillary (A), cribriform (B), and solid (C) structures that show a higher proportion of cytoplasmic and membranous MUC1 expression than seen among other carcinoma cells that do not include to the putative anoikis‐resistant structures (red arrows). The latter cells are mainly non‐layered carcinoma cells forming gland‐like structures. In the stroma, there are occasional positively stained plasma cells. Scale bars 100 μm.

**FIGURE 2:**
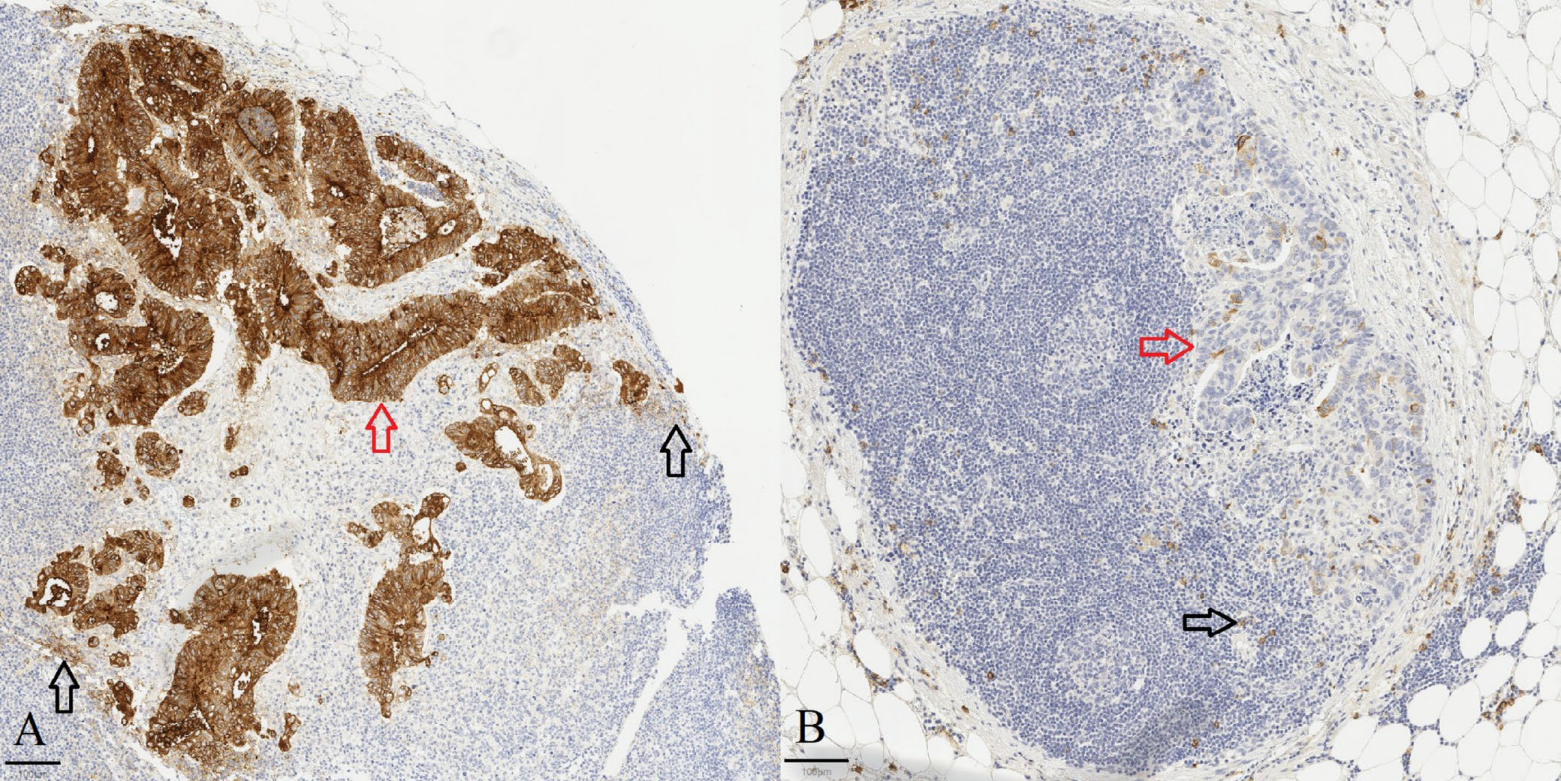
Examples of MUC1 (E29) immunohistochemistry in colorectal carcinoma lymph node metastases in cases with and without distant metastasis. In (A), a case with distant metastasis shows cytoplasmic expression in nearly all carcinoma cells (red arrow). In contrast, in (B), a case without distant metastasis, the proportion of positive cells is low (red arrow). Black arrows show internal positive staining control of plasma cells and other lymphatic cells. Scale bars 100 μm.

Cytoplasmic and membranous staining of MUC1 was separately assessed. For both subcellular locations, the proportion (0%–100%) of positively stained carcinoma cells was assessed including cells with weak intensity (Figures 1 and 2). The proportion of positive staining was assessed for all carcinoma cells (including both putative AR cells and other carcinoma cells), and separately for MIPs, cribriform structures and solid structures. For the assessment of interobserver agreement of the recognition of MUC1 staining in different carcinoma cell subpopulations, two subsets formed of 10 cases were independently studied by two observers (T.T.M. and T.J.K.), and for intraobserver repeatability by one observer (T.J.K.).

### 2.5 Proliferation and Apoptosis in Putative AR Structures

Apoptosis and proliferation indices in primary tumors were evaluated with M30 and Ki‐67 immunohistochemistry, respectively [28, 32]. M30 immunohistochemistry specifically detects the caspase‐cleaved form of cytokeratin 18, providing a sensitive marker for early apoptotic events in epithelial cells [37]. MIPs, cribriform structures, solid structures, and other cancer cells were separately assessed as described previously [32].

### 2.6 Statistical Analysis

For statistical analysis, we used IBM SPSS Statistics, version 28 (IBM Corp., Armonk, NY, USA). A two‐tailed, exact *p* < 0.05 was considered statistically significant. As the distributions of MUC1 positive cells in carcinoma cells were skewed, we applied nonparametric tests including Mann–Whitney or Kruskal–Wallis tests to assess their relationship with the clinicopathological factors. Similarly, nonparametric Spearman rank correlation and Wilcoxon matched‐pair tests were used for assessing associations between the variables, and for comparing proportions of MUC1 expressing cells in metastases and primary tumors, respectively. We determined the optimal cutoff values for the proportions of MUC1 positive cells in survival analyses using the receiver operating characteristics (ROC) curve and the Youden index [38]. For univariate survival analysis, we created Kaplan–Meier curves for cancer‐specific survival (CSS) and the log‐rank test was used to evaluate the statistical significance of differences in the survival plots. Univariate and bivariate Cox regression models were used to analyze the independent prognostic effect of the proportions on CSS when adjusted for covariates. Because of the low number of cases for proper multivariate analyses, we used models for one covariate at a time, as described in previous literature [32, 39, 40]. For interobserver agreement, the Pearson correlation coefficient was used.

## 3 Results

Histological, immunohistochemical, molecular pathological, and clinical characteristics of the cases including areal densities of the putative AR structures in primary tumors and nodal metastasis have been reported previously [28, 32].

### 3.1 Assessment of MUC1 Expression in Different Populations of Carcinoma Cells

MUC1 staining located in carcinoma cells, and stromal plasma cells served as an internal positive control [41, 42] (Figures 1 and 2). In addition to overall staining, all three types of putative AR structures (micropapillary, cribriform, and solid) could be assessed in MUC1‐stained sections across most cases (Figures 1 and 2). In all carcinoma cells of primary tumors, there was a high correlation between cytoplasmic and membranous expression (Spearman's rho = 0.675; *p* < 0.001). In some cases with intense cytoplasmic staining, the cytoplasmic staining hampered the assessment of membranous staining. Cells with a strong homogeneous staining extending from the cytoplasm to the cell membrane and without any accentuation of the cell membrane were classified as cytoplasm positive and membrane positive. No differences in MUC1 expression were observed between cases treated with preoperative radiation therapy and untreated cases, either in primary tumors or lymph node metastases (*p* = 0.075–0.656).

Interobserver agreement was primarily evaluated by scoring 10 randomly selected cases (T.T.M. and T.J.K.) for MUC1 expression in different subpopulations of carcinoma cells. Pearson correlation coefficients were as follows—all carcinoma cells: cytoplasmic 0.845; membranous 0.599; MIPs: cytoplasmic −0.082; membranous 0.820; cribriform structures: cytoplasmic 0.911; membranous 0.808; solid structures: cytoplasmic 0.784; membranous 0.377. Although the interobserver correlation was generally moderate or high, it was noted that identification of MIPs and solid structures in immunostained slides requires experience. For these two structures, interobserver agreement was tested (T.T.M. and T.J.K.) with another 10 cases where MIPs and solid structures had been annotated for scoring (T.T.M.), resulting in the following Pearson correlations: MIPs: cytoplasmic 0.883; membranous 0.791; solid structures: membranous 0.977; and cytoplasmic 0.971. Finally, intraobserver agreement was studied by one observer (T.J.K.) in 10 cases, showing strong Pearson correlations: for all carcinoma cells cytoplasmic 0.923; membranous 0.934; for MIPs: cytoplasmic 0.878; membranous 0.904; for cribriform structures: cytoplasmic 0.973; membranous 0.784; and for solid structures: cytoplasmic 0.985; membranous 0.944.

### 3.2 MUC1 Expression: Comparison of Subpopulations and Relationship With Their Areal Densities

Cribriform and solid structures had more abundant cytoplasmic and membranous MUC1 than overall carcinoma cells, while MIPs resembled overall carcinoma cells (Table 2). In contrast, the areal densities of putative AR structures (MIPs, cribriform, and solid), alone or combined (Total AR; data not shown), or their densities in nodal metastases did not correlate with MUC1 expression (*p* = 0.118–0.980). However, cases with a high Total AR areal density (> 6.86/mm^2^) showed elevated cytoplasmic MUC1 in overall carcinoma cells, MIPs, and cribriform structures (*p* = 0.026–0.048), but not in solid structures (Tables S1 and S2). MUC1 may thus be more important in specific AR subpopulations. Membranous MUC1 was not associated with Total AR level (Table S2).

**TABLE 2:** Comparison of MUC1 cytoplasmic and membranous expression in all carcinoma cells and in putative anoikis‐resistant (AR) subpopulations of carcinoma cells.

Subcellular location and cell type	Percent positive cells			Difference in the proportion (%) of positive cells			*N*	*Z*	*p*
	Median	Mean	IQR	Median	Mean	IQR			
**Cytoplasmic MUC1**									
All carcinoma cells	80.0	70.1	50.0–96.3				118		
MIPs	82.5	67.4	40.0–100	0	0.4	−5.0‐10.0	94	−0.487	0.626
Cribriform structures	85.0	72.2	58.8–100	0	3.3	0.0–6.3	114	−3.617	< 0.001
Solid structures	95.0	83.3	80.0–100	0	7.4	0.0–15.0	75	−3.551	< 0.001
**Membranous MUC1**									
All carcinoma cells	20.0	24.6	10.0–35.0				118		
MIPs	20.0	26.7	3.8–40.0	0	0.4	−5.0‐11.3	94	−1.608	0.108
Cribriform structures	20.0	27.0	10.0–35.0	0	2.9	0.0–6.3	114	−3.004	0.003
Solid structures	25.0	35.3	10.0–60.0	0	5.7	−5.0‐15.0	75	−2.028	0.043

*Note:* Difference refers to the difference in the positive cell proportion between expression in all carcinoma cells and that in each type of putative AR structures. Statistical significance of the difference between all carcinoma cells and each type of the putative AR subpopulations was assessed with the Wilcoxon signed‐rank test. *Z* is the test statistic.

Abbreviation: IQR, interquartile range.

### 3.3 MUC1 in Primary Tumors: Relationship With Cell Proliferation and Apoptosis Rates

Cytoplasmic MUC1 in all carcinoma cells or AR subpopulations was not associated with apoptosis or proliferation rates (M30 or Ki‐67 positivity; *p* = 0.256–0.856; Spearman). Membranous MUC1 also showed no link to M30 (*p* = 0.491–0.92). However, membranous MUC1 weakly correlated with Ki‐67 in all carcinoma cells (*ρ* = 0.215, *p* = 0.024) and solid structures (*ρ* = 0.349, *p* = 0.011), but not in MIPs (*ρ* = −0.027, *p* = 0.814) or cribriform structures (*ρ* = 0.169, *p* = 0.087).

### 3.4 MUC1 in Primary Tumors: Association With Clinicopathologic Features and Prognosis

Association of MUC1 expression and clinicopathological features is summarized in Tables S1, S2. MMR deficiency was associated with high cytoplasmic MUC1 expression in MIPs, cribriform structures, and all carcinoma cells. Cytoplasmic MUC1 positivity in cribriform structures was associated with *BRAF mutation* and with serrated cancer type. Proximal location and high WHO grade were associated with MUC1 expression in MIPs and cribriform structures. Membranous MUC1 positivity was associated with female gender, high WHO grade, lymphatic invasion, *BRAF mutation*, pushing border, and proximal primary tumor location. In contrast, cytoplasmic MUC1 positivity in solid structures did not associate with any clinicopathological factors, and the sole association of higher membranous MUC1 staining in solid structures was with the tumor having a non‐infiltrating (i.e., pushing) border. Neither cytoplasmic nor membranous MUC1 in primary tumors had prognostic impact, whether analyzed in the entire cohort or in stages I–II versus III–IV, and whether assessed in all carcinoma cells, MIPs, cribriform, or solid structures.

### 3.5 Comparison of MUC1 Expression in Primary Tumors and in Lymph Node Metastases

**FIGURE 3:**
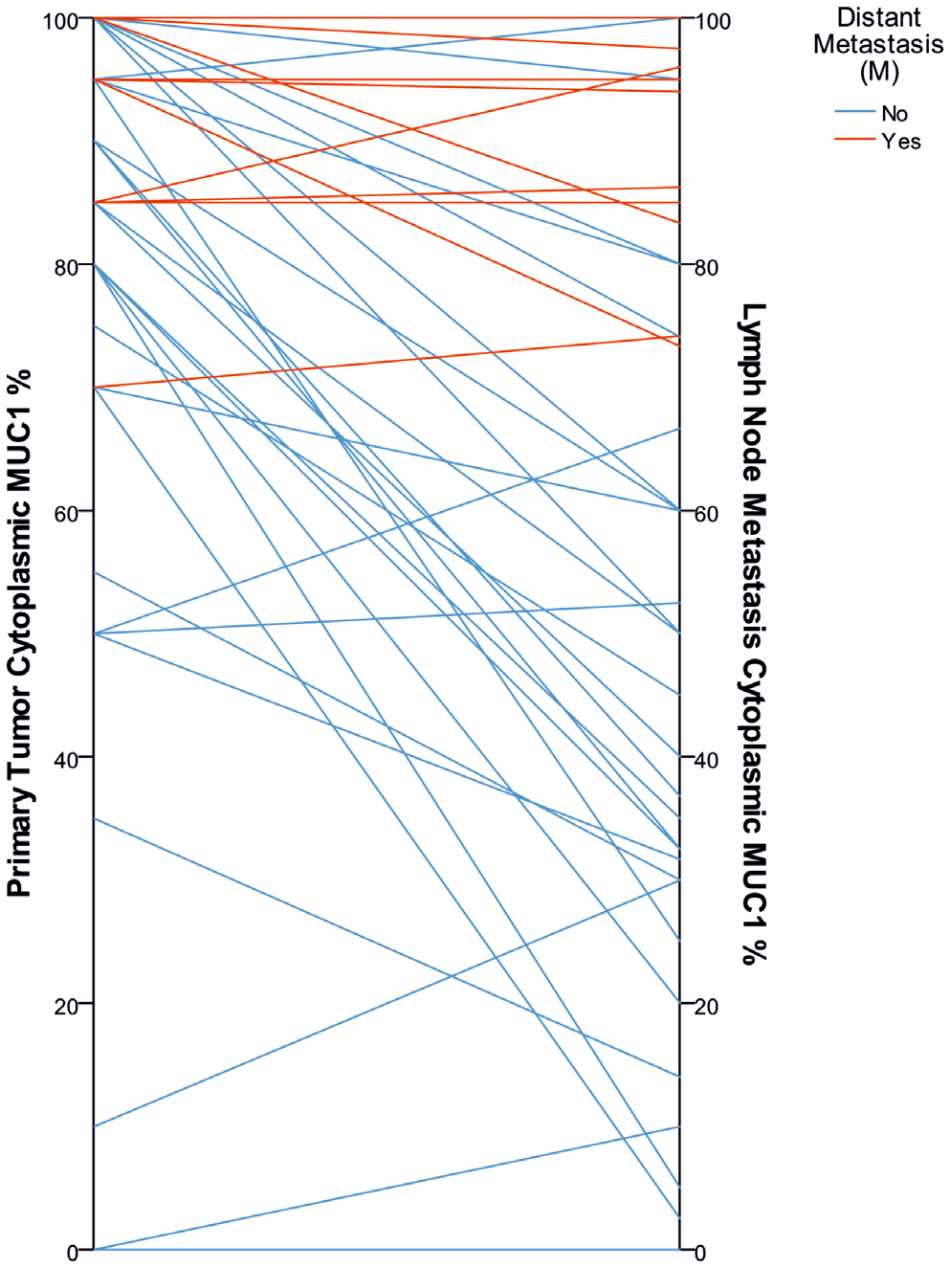
Parallelogram showing proportions of cytoplasmic MUC1 expression in all carcinoma cells in primary tumors and corresponding lymph node metastases (*n* = 46). Cases with (red) and without (blue).

MUC1 expression in nodal metastases and corresponding primary tumors correlated positively, more strongly for cytoplasmic than membranous MUC1 (Table S3). Lymph node metastases generally showed lower membranous and cytoplasmic MUC1 (Table 3). However, some cases retained or even increased cytoplasmic MUC1 positivity (Figure 3).

**TABLE 3:** Comparison of MUC1 expression in carcinoma cells in primary CRC tumors with lymph node metastases (N+) and in lymph node (LN) metastases.

	Primary Tumor (N+) proportion of positive cells median (IQR)	LN metastasis proportion of positive cells median (IQR)	*p* (Primary tumor [N+] vs. LN metastasis)
Cytoplasmic MUC1			
All carcinoma cells	85 (67.5–95)	51.3 (30.0–84.6)	< 0.001
MIPs	95 (55–100)	65 (19.5–95.0)	0.027
Cribriform structures	85 (65–100)	51.0 (30.0–91.7)	< 0.001
Solid structures	92.5 (85–100)	69.2 (36.3–90.6)	< 0.001
Membranous MUC1			
All carcinoma cells	20 (10–37.5)	10.0 (3.33–25.0)	0.002
MIPs	30 (5–45)	10.0 (0–30.0)	0.006
Cribriform structures	25 (10–30)	8.44 (3.00–22.2)	0.001
Solid structures	22.5 (10–47.5)	6.37 (1.67–29.6)	< 0.001

*Note:* Median proportions of positively stained carcinoma cells with interquartile range (IQR) for membranous or cytoplasmic MUC1 among all carcinoma cells and among the putative AR structure types are shown. *p*‐values are presented for the Wilcoxon signed‐rank test.

### 3.6 Prognostic Effect of MUC1 Expression in Lymph Node Metastases

**FIGURE 4:**
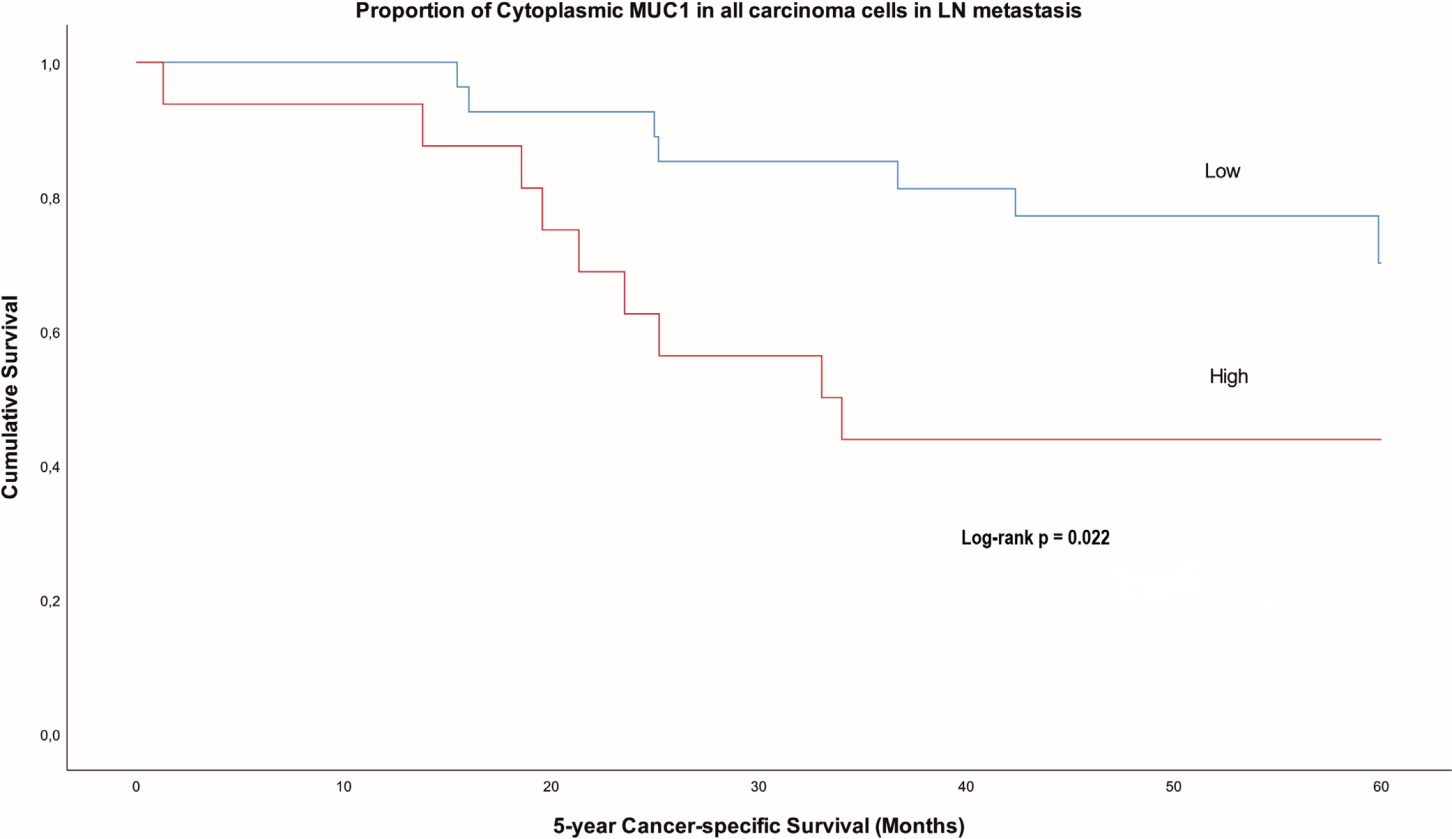
Kaplan‐Meier curve showing 5‐year cancer‐specific survival (60 months) in patients (n=46) with high (n=17) or low (n=29) proportion of cytoplasmic MUC1 expression in all carcinoma cells in lymph node metastases (cut‐off 73.7%).

In lymph node metastases, high cytoplasmic MUC1 expression is associated with adverse prognosis, reaching significance in univariate analysis for all carcinoma cells (Figure 4, cutoff 73.7%, *p* = 0.022) and solid structures (cutoff 75.7%, *p* = 0.013), but not for MIPs (*p* = 0.062) or cribriform structures (*p* = 0.306). In multivariate models, high cytoplasmic MUC1 (> 73.7%) in nodal metastasis remained an independent prognostic factor for a low 5‐year cancer‐specific survival in all models except the model with distant metastasis (M), indicating its potential utility as an independent prognostic factor (Table 4). Conversely, membranous MUC1 in nodal metastases showed no prognostic impact (all carcinoma cells *p* = 0.125, MIPs *p* = 0.228, cribriform *p* = 0.209, and solid *p* = 0.187).

**TABLE 4:** Univariate and multivariate Cox regression model for 5‐year cancer‐specific survival evaluating the prognostic independence of high cytoplasmic MUC1 expression of all carcinoma cells in lymph node metastasis.

Covariates	Univariate HR (95% CI)	Model 1	Model 2	Model 3	Model 4	Model 5	Model 6	Model 7
		Total AR HR (95% CI)	T HR (95% CI)	N1‐2 HR (95% CI)	M HR (95% CI)	ENE HR (95% CI)	Grade HR (95% CI)	Location HR (95% CI)
	*p*	*p*	*p*	*p*	*p*	*p*	*p*	*p*
Lymph node metastasis Cytoplasmic MUC1 > 73.7%	**3.68** **(1.22–11.1)** ***p* = 0.021**	**4.14** **(1.37–12.5)** ***p* = 0.012**	**2.99** **(1.10–8.13)** ***p* = 0.032**	**3.11** **(1.14–8.52)** ***p* = 0.027**	1.67 (0.48–5.80) *p* = 0.420	**3.56** **(1.28–9.90)** ***p* = 0.015**	**3.03** **(1.09–8.40)** ***p* = 0.034**	**3.03** **(1.11–8.28)** ***p* = 0.031**
Primary Tumor Total AR > 6.86/mm^2^	1.82 (0.58–5.66) *p* = 0.302	3.16 (0.90–11.2) *p* = 0.074						
T Stage T1‐2 versus T3‐4	0.69 (0.20–2.43) *p* = 0.565		0.78 (0.22–2.75) *p* = 0.695					
N Stage N1 versus N2	1.70 (0.63–4.57) *p* = 0.293			1.76 (0.65–4.75) *p* = 0.265				
Distant metastasis (M)	**4.82** **(1.72–13.5)** ***p* = 0.003**				3.50 (0.98–12.5) *p* = 0.053			
Extranodal extension (ENE)	2.13 (0.74–6.15) *p* = 0.163					2.61 (0.88–7.72) *p* = 0.082		
WHO Grade 1–2 versus 3	1.37 (0.44–4.25) *p* = 0.587						1.02 (0.32–3.27) *p* = 0.972	
Location (rectum/colon)	0.85 (0.32–2.26) *p* = 0.738							0.97 (0.36–2.63) *p* = 0.958

*Note:* In Models 1–7, High Cytoplasmic MUC1 (> 73.7%) in Lymph node metastasis was assessed with individual covariate adjustments due to the limited number of cases for multivariate analysis. Model 1: Primary Tumor Total AR density > 6.86/mm^2^ (density of micropapillary, cribriform, and solid structures per mm^2^); Model 2: T stage (T1‐2 vs. T3‐4); Model 3: N Stage (N1 vs. N2); Model 4: Distant Metastasis (M0 vs. M1); Model 5: Extranodal extension in any lymph node metastasis; Model 6: WHO Grade (1–2 vs. 3); Model 7: Location (Rectum vs. Colon). Bold values indicate statistically significant *p* values. Univariate column presents the crude value.

Abbreviation: HR, Hazard ratio.

### 3.7 MUC1 in Primary Tumors and Lymph Node Metastases and the Presence of Distant Metastases

**FIGURE 5:**
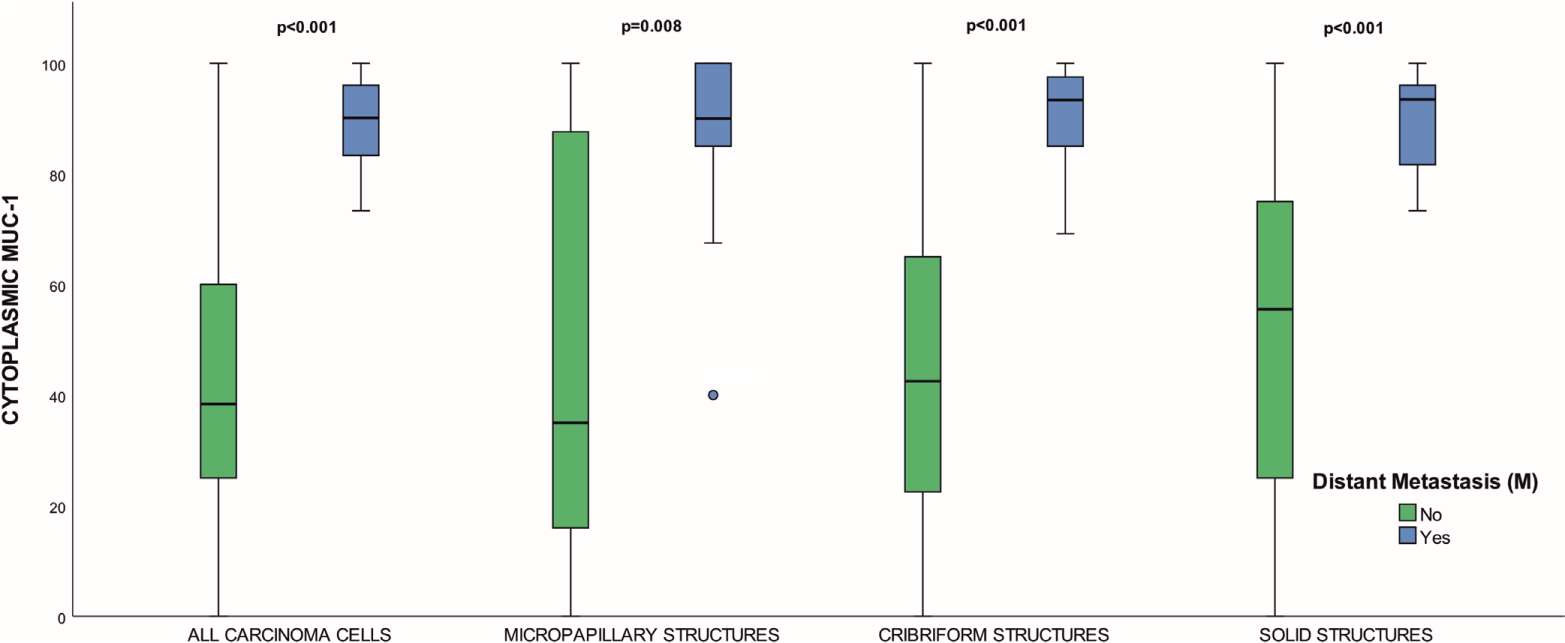
Comparison of cytoplasmic MUC1 expression (proportion of positive cells, 0%–100%) in lymph node metastases between cases with (*n* = 10) and without distant metastasis (*n* = 36). Comparison includes subpopulations of all carcinoma cells (putative anoikis‐resistant and non‐anoikis‐resistant cells) and putative anoikis‐resistant cells in micropapillary (MIPs), cribriform, and solid structures. Comparison between cases with and without distant metastasis was made with the Mann–Whitney *U* test. Range (whiskers), interquartile range (box), and median (horizontal line) are shown.

In primary tumors, cytoplasmic or membranous MUC1 did not significantly differ between cases with (*n* = 16) and without (*n* = 101) distant metastases, whether analyzing all carcinoma cells or putative AR structures (*p* = 0.168–0.902). However, in nodal‐metastatic cases (*n* = 46), nodal MUC1 was higher in those who had developed distant metastases (*n* = 10). In nodal carcinoma cells, the presence of distant metastasis correlated with elevated cytoplasmic (all carcinoma cells *p* < 0.001, MIP, *p* = 0.008, cribriform, *p* < 0.001, solid *p* < 0.001; Figure 5) and membranous MUC1 expression (all carcinoma cells, *p* = 0.005, MIP, *p* = 0.012, cribriform, *p* = 0.003, and solid, *p* = 0.028).

**FIGURE 6:**
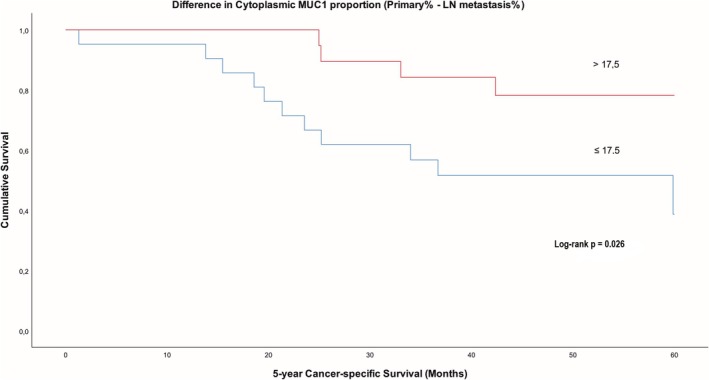
Kaplan–Meier curve showing cancer‐specific survival (60 months) in patients (*n* = 46) with high (*n* = 22) and low (*n* = 24) difference (= primary tumor value—lymph node [LN] metastasis value) in cytoplasmic MUC1 expression in all carcinoma cells between primary tumor and lymph node metastases (cut‐off 17.5%).

Notably, patients without distant metastases often showed reduced lymph node MUC1 relative to primary tumors (Figure 3). A reduction of > 17.5% in cytoplasmic MUC1 between primary and nodal sites predicted better survival (*p* = 0.026; Figure 6). Overall, persistently high MUC1 from primary tumors to nodal metastases may indicate worse prognosis and increased risk of distant metastases.

## 4 Discussion

MUC1 is a glycoprotein implicated in anoikis inhibition and as a biomarker of adverse prognosis [12, 13, 14, 15, 16, 17, 18, 19, 20]. We analyzed MUC1 expression in CRC primary tumors and lymph node metastases to investigate its role in the formation of putative AR structures and prognostic significance. Except for MIPs, these AR structures showed overexpression of MUC1, suggesting an involvement of MUC1 in their biology. While MUC1 in primary tumors had no prognostic impact, high MUC1 in nodal metastases was associated with the presence of distant metastasis and adverse prognosis, supporting its value as a prognostic biomarker in stage III CRC [6].

We previously described a method to identify putative AR subpopulations of carcinoma cells in conventional tissue sections. These subpopulations include inner cells in cribriform and solid structures and suprabasal cells in micropapillary structures (MIPs), all of which lack contact with the mesenchymal areas or basement membrane proteins but still do not show an increased apoptosis rate [28, 32]. It is important to note that MIPs in our study do not refer to the traditional micropapillary growth pattern or micropapillary carcinoma, but rather to a specific histological feature defined as cell stacks at the luminal side of glandular structures. In this study, we detected MUC1 overexpression in two of the putative AR subtypes, cribriform and solid structures, and observed that a high areal density (> 6.86/mm^2^) of putative AR structures is associated with higher levels of cytoplasmic MUC1 expression in MIPs, cribriform structures, and across all carcinoma cells. These findings suggest that MUC1 is involved in the pathogenesis of the putative AR structures and that they are also consistent with the concept that MUC1 has a role in anoikis resistance [13, 14]. However, MUC1 expression did not correlate with apoptosis rates in these populations indicating that there is no direct quantitative relationship between MUC1 expression and anoikis inhibition.

Given the potential importance of subcellular localization of MUC1 [13, 14, 30], we analyzed cytoplasmic and membranous MUC1 expression separately. Distinguishing membranous staining was difficult in cases with strong cytoplasmic staining, making it challenging to assess subcellular location and polarity of the staining. Our analysis showed that cytoplasmic MUC1 staining, rather than membranous staining, was more strongly associated with poor prognosis, indirectly suggesting that intracellular localization may play a role in mediating anoikis resistance. This contrasts with in vitro findings, where the extracellular domain, and the loss of normal apical polarity in MUC1 expression contribute to anoikis resistance [13, 14, 30]. To more precisely evaluate the role of membrane‐associated MUC1 staining and the importance of MUC1 expression polarity in anoikis inhibition, adjustment of the staining intensity or double staining techniques with cell membrane and apical/basal markers would be required.

MUC1 expression in primary tumors did not correlate with prognosis, contrary to many prior studies linking high MUC1 to worse CRC outcomes [12, 13, 14, 15, 17, 18, 19, 20]. Discrepancies may stem from differing IHC methods, antibodies, and cutoff definitions. We used a clinically validated antibody [36], included weak staining as positive to avoid diagnostic drift and simplify scoring [43], and employed ROC analysis to define optimal cutoffs. A notable methodological difference compared to most previous studies is the detection system used in immunohistochemistry. Unlike biotin‐based systems in the majority of earlier studies [17, 18], our polymer‐based, biotin‐free method avoids nonspecific staining from endogenous biotin present in normal and neoplastic colon and many other tissues [21, 44, 45]. Since endogenous biotin serves as a cofactor in various metabolic enzymes, it might even have prognostic value on its own. Methodological refinements may explain our divergent findings and underscore the need for robust IHC protocols in prognostic research.

MUC1 expression in primary tumors correlated with that in metastases, aligning with findings for other CRC biomarkers [5]. This suggests that the metastatic clones mostly retain expression patterns of the primary tumor. Similarly, our previous study indicated that areal densities of the putative AR structures positively correlated between the primary tumor and metastasis; however, there was a significantly increased density in nodal metastasis [28, 32]. Such enrichment of the putative AR structures in the metastatic site is consistent with the concept that anoikis resistance is necessary for metastasis formation and therefore may be a basis for clonal selection leading to a more abundant formation of putative AR structures at the metastatic site [28, 32]. Still, MUC1 levels were generally lower in metastases, suggesting that high MUC1 expression may not be a prerequisite for the formation of metastasis or a dominant factor in anoikis resistance. Experimental data, such as from pancreatic cancer models [46], are needed to clarify MUC1's exact role in CRC.

High MUC1 expression in lymph node metastases was associated with the presence of distant metastasis and adverse prognosis. Previous analyses of prognostic biomarkers in nodal metastases are rare. Karamitopoulou et al. [6] reported a similar association with prognosis in CRC. Our findings suggest that high MUC1 expression in nodal metastasis could potentially serve as a marker for the risk of subsequent stage IV disease in CRC with nodal metastasis. Cases with distant metastases typically showed > 80% nodal metastasis MUC1 positivity, versus ≤ 60% in those without (Figure 5). Additionally, the difference in MUC1 expression between the primary tumor and lymph node metastasis was related to metastatic status and survival, with a reduction in expression in nodal metastasis in cases without distant metastasis and better prognosis. We speculate that in addition to the selection of possibly highly aggressive tumor cell clones along with the capacity for anoikis resistance, selection might also involve other, less aggressive properties of the cells.

About half of CRC distant metastases may originate from lymph node metastases [47]. Persistent MUC1 overexpression in both primary and nodal sites could indicate a competitive subclone prone to further dissemination. If MUC1 actively promotes metastasis, it could be a target for antibody‐based therapies [46] or vaccine approaches [48]. Though our cross‐sectional design does not confirm predictive value for future distant spread or guide adjuvant therapy decisions in stage III CRC [49], these findings highlight the need for further investigation.

Limitations of this study include the relatively small size of the cohort of 118 cases, of which 52 had nodal metastasis and 46 with available sample material. Therefore, these observations should be verified in larger independent cohorts. Additionally, although the general quality of MUC1 immunohistochemistry was high, further optimization is needed to better distinguish between cytoplasmic and membranous staining and to observe cell polarity. A potential solution could be the use of double staining with an apical or basal marker alongside MUC1.

## 5 Conclusions

Putative AR structures in CRC overexpress MUC1. While MUC1 in primary tumors did not show prognostic value or association with metastasis, consistently high MUC1 expression both in primary tumors and nodal metastases was associated with adverse prognosis. Additionally, high MUC1 expression in nodal metastasis was associated with distant metastasis. These structural and clinical correlations support the role of MUC1 in anoikis resistance but need experimental evidence. The identification of MUC1 in nodal metastatic cells as a prognostic biomarker contrasts with its inconclusive role in primary tumors and suggests that future prognostic studies of MUC1 should focus on lymph node metastatic tissue.

## Funding

This work was supported by the Thelma Mäkikyrö Foundation.

## Ethics Statement

This research project received approval from the Ethics Committee of Oulu University Hospital (58/2005, 184/2009).

## Consent

Informed consent was obtained from all individual participants included in the study.

## Conflicts of Interest

The authors declare no conflicts of interest.

## Supporting information


**Table S1:** apm70105‐sup‐0001‐TableS1.docx.

**Table S2:** apm70105‐sup‐0002‐TableS2.docx.

**Table S3:** apm70105‐sup‐0003‐TableS3.docx.

## Data Availability

The authors have nothing to report.
