# Supplementary material for: MUC1 in Colorectal Carcinoma: Association With Prognosis and Putative Anoikis‐Resistant Structures
Source: APMIS. 2025 Dec 2;133(12):e70105. doi: 10.1111/apm.70105 (PMC12673294; doi:10.1111/apm.70105)
Supplement: Supplementary file 1 — Table S1: apm70105‐sup‐0001‐TableS1.docx. [file APM-133-0-s003.docx]

**Supplementary Table 1.** Relationship between clinicopathological features and the proportion of cells with cytoplasmic MUC1 expression in the putative anoikis-resistant populations (MIPs, cribriform, and solid) and all carcinoma cells in primary tumors.

|  | MIP  Cytoplasmic MUC1  Median (IQR) | Cribriform  Cytoplasmic MUC1  Median (IQR) | Solid  Cytoplasmic MUC1  Median (IQR) | All carcinoma cells  Cytoplasmic MUC1  Median (IQR) |
| --- | --- | --- | --- | --- |
| Total AR | **p=0.048** | **p=0.026** | p=0.423 | **p=0.026** |
| High (>6.86/mm^2^) | 100 (75-100) | 90 (85-100) | 95 (90-100) | 85 (85-95) |
| Low | 75 (35-100) | 85 (55-100) | 95 (70-100) | 75 (45-95) |
| Age | p=0.415 | p=0.451 | p=0.793 | p=0.554 |
| > 65 | 85 (50-100) | 85 (65-100) | 95 (85-100) | 80 (65-95) |
| ≤ 65 | 75 (15-100) | 80 (55-100) | 95 (70-100) | 82.5 (47.5-97.5) |
| Sex | **p=0.036** | p=0.075 | p=0.418 | p=0.089 |
| Male | 60 (25-100) | 82.5 (50-95) | 95 (70-100) | 77.5 (40-95) |
| Female | 97.5 (65-100) | 90 (60-100) | 95 (85-100) | 85 (70-100) |
| Primary tumor location | **p=0.012** | **p=0.014** | p=0.329 | p=0.062 |
| Proximal | 100 (70-100) | 95 (70-100) | 100 (85-100) | 90 (65-100) |
| Distal | 67.5 (25-100) | 85 (80-100) | 100 (85-100) | 85 (75-95) |
| Rectum | 65 (15-100) | 72.5 (50-95) | 90 (80-100) | 75 (45-90) |
| Preoperative therapy (rectal) | **p=0.541** | **p=0.794** | **p=0.914** | **p=0.749** |
| Yes | 75 (30-100) | 65 (55-92.5) | 95 (70-100) | 70 (50-95) |
| No | 60 (15-100) | 85 (45-95) | 90 (90-100) | 75 (30-90) |
| WHO Grade | **p=0.044** | **p=0.016** | p=0.192 | p=0.077 |
| G1 Well differentiated | 62.5 (25-100) | 57.5 (32.5-80) | 100 (70-100 | 65 (35-85) |
| G2 Moderately differentiated | 75 (40-100) | 85 (60-100) | 90 (80-100) | 80 (55-95) |
| G3 Poorly differentiated | 100 (100-100) | 100 (75-100) | 100 (95-100) | 95 (80-100) |
| TNM Stage | p=0.818 | p=0.553 | p=0.905 | p=0.582 |
| Stage I | 85 (25-100) | 85 (60-100) | 95 (90-100) | 85 (65-100) |
| Stage II | 65 (40-100) | 82.5 (42.5-100) | 97.5 (60-100) | 75 (40-97.5) |
| Stage III | 95 (55-100) | 85 (65-95) | 90 (85-100) | 85 (55-95) |
| Stage IV | 92.5 (45-100) | 92.5 (80-100) | 97.5 (75-100) | 85 (72.5-97.5) |
| Metastasis (M) | p=0.742 | p=0.168 | p=0.902 | p=0.355 |
| Yes | 92.5 (45-100) | 92.5 (80-100) | 97.5 (75-100) | 85 (72.5-97.5) |
| No | 75 (40-100) | 85 (55-100) | 95 (85-100) | 80 (50-95) |
| Lymph node metastasis | p=0.335 | p=0.526 | p=0.701 | p=0.287 |
| Yes | 95 (55-100) | 85 (65-100) | 92.5 (85-100) | 85 (67.5-95) |
| No | 70 (40-100) | 85 (52.5-100) | 95 (75-100) | 75 (45-100) |
| Extranodal extension | p=0.899 | p=0.965 | p=0.917 | p=0.769 |
| Yes | 90 (60-100) | 87.5 (60-100) | 95 (90-100) | 85 (60-97.5) |
| No | 95 (40-100) | 90 (77.5-95) | 95 (90-100) | 85 (80-95) |
| Lymphatic invasion | p=0.154 | p=0.223 | p=0.898 | p=0.246 |
| Yes | 90 (55-100) | 87.5 (70-100) | 95 (90-100) | 85 (70-95) |
| No | 70 (32.5-100) | 85 (45-100) | 95 (60-100) | 75 (40-100) |
| Blood vessel invasion | p=0.615 | p=0.527 | p=0.453 | p=0.762 |
| Yes | 85 (40-100) | 85 (60-95) | 95 (85-100) | 85 (60-90) |
| No | 80 (40-100) | 85 (60-100) | 95 (80-100) | 80 (50-100) |
| Infiltrating border | p=0.463 | p=0.776 | p=0.238 | p=0.604 |
| Yes | 85 (65-100) | 85 (55-95) | 90 (70-100) | 82.5 (47.5-92.5) |
| No | 80 (40-100) | 85 (60-100) | 95 (90-100) | 80 (50-100) |
| Cancer type | p=0.309 | **p=0.044** | p=0.559 | p=0.176 |
| Conventional | 75 (35-100) | 85 (55-100) | 95 (80-100) | 80 (50-95) |
| Serrated | 90 (65-100) | 95 (70-100) | 97.5 (90-100) | 85 (70-100) |
| Mismatch repair (MMR) | **p=0.019** | **p=0.010** | p=0.094 | **p=0.009** |
| Proficient | 75 (40-100) | 85 (55-100) | 95 (80-100) | 80 (50-95) |
| Deficient | 100 (100-100) | 100 (100-100) | 100 (100-100) | 100 (95-100) |
| *BRAF* mutation | p=0.109 | **p=0.030** | p=0.956 | p=0.107 |
| Yes | 100 (80-100) | 100 (85-100) | 92.5 (87.5-100) | 95 (75-100) |
| No | 75 (40-100) | 85 (55-100) | 95 (80-100) | 80 (50-95) |
| *KRAS* mutation | p=0.456 | p=0.341 | p=0.231 | p=0.623 |
| Yes | 75 (50-100) | 85 (55-100) | 100 (87.5-100) | 85 (50-100) |
| No | 75 (25-100) | 85 (60-95) | 95 (70-100) | 80 (50-95) |

P-values are presented for Mann-Whitney U or Kruskall-Wallis tests.
