# Supplementary material for: MUC1 in Colorectal Carcinoma: Association With Prognosis and Putative Anoikis‐Resistant Structures
Source: APMIS. 2025 Dec 2;133(12):e70105. doi: 10.1111/apm.70105 (PMC12673294; doi:10.1111/apm.70105)
Supplement: Supplementary file 2 — Table S2: apm70105‐sup‐0002‐TableS2.docx. [file APM-133-0-s002.docx]

**Supplementary Table 2.** Relationship between clinicopathological features and the proportion of carcinoma cells with membranous MUC1 expression in different putative anoikis-resistant populations (MIPs, cribriform, and solid) and in all carcinoma cells.

|  | MIP  Membranous MUC1  Median (IQR) | Cribriform  Membranous MUC1  Median (IQR) | Solid  Membranous MUC1  Median (IQR) | All carcinoma cells  Membranous MUC1  Median (IQR) |
| --- | --- | --- | --- | --- |
| Total AR | p=0.130 | p=0.447 | p=0.980 | p=0.638 |
| High (>6.86/mm^2^) | 30 (20-35) | 22.5 (12.5-32.5) | 22.5 (15-50) | 20 (10-35) |
| Low | 15 (0-40) | 17.5 (10-35) | 25 (10-65) | 20 (10-35) |
| Age | p=0.813 | p=0.317 | p=0.748 | p=0.741 |
| > 65 | 20 (5-35) | 25 (10-40) | 25 (10-70) | 20 (10-35) |
| ≤ 65 | 20 (0-55) | 15 (10-30) | 25 (10-50) | 20 (10-37.5) |
| **Sex** | **p=0.036** | p=0.129 | p=0.405 | p=0.343 |
| Male | 15 (0-35) | 15 (10-35) | 20 (10-50) | 15 (10-40) |
| Female | 25 (10-45) | 25 (10-40) | 30 (15-65) | 22.5 (10-35) |
| Primary tumor location | **p=0.026** | p=0.165 | p=0.498 | p=0.324 |
| Proximal | 25 (20-45) | 25 (10-57.5) | 25 (15-80) | 15 (10-40) |
| Distal | 22.5 (5-45) | 28 (12.5-35) | 30 (15-55) | 25 (15-40) |
| Rectum | 10 (0-35) | 15 (5-30) | 20 (10-50) | 15 (10-32.5) |
| Preoperative therapy (rectal) | p=0.675 | p=0.380 | p=0.468 | p=0.806 |
| Yes | 10 (0-30) | 15 (5-35) | 25 (10-70) | 15 (0-35) |
| No | 12.5 (0-37.5) | 15 (7.5-25) | 20 (10-35) | 20 (10-30) |
| WHO Grade | p=0.272 | **p=0.037** | p=0.710 | **p=0.042** |
| G1 Well differentiated | 15 (0-37.5) | 10 (5-15) | 35 (20-40) | 10 (0-15) |
| G2 Moderately differentiated | 20 (5-35) | 25 (10-35) | 25 (10-57.5) | 20 (10-35) |
| G3 Poorly differentiated | 35 (17.5-67.5) | 32.5 (15-80) | 27.5 (15-80) | 25 (10-80) |
| TNM Stage | p=0.608 | p=0.927 | p=0.227 | p=0.735 |
| Stage I | 15 (0-40) | 20 (10-35) | 55 (20-77.5) | 20 (10-30) |
| Stage II | 15 (5-35) | 25 (2.5-40) | 25 (10-60) | 15 (5-37.5) |
| Stage III | 30 (5-45) | 25 (10-35) | 25 (10-50) | 20 (10-40) |
| Stage IV | 22.5 (2.5-57.5) | 15 (10-25) | 15 (5-35) | 20 (7.5-32.5) |
| Metastasis (M) | p=0.603 | p=0.575 | p=0.241 | p=0.933 |
| Yes | 22.5 (2.5-57.5) | 15 (10-25) | 15 (5-35) | 20 (7.5-32.5) |
| No | 20 (5-40) | 25 (10-40) | 30 (10-60) | 20 (10-35) |
| Lymph node metastasis | p=0.072 | p=0.643 | p=0.239 | p=0.192 |
| Yes | 30 (5-40) | 25 (10-30) | 22.5 (10-47.5) | 20 (10-37.5) |
| No | 15 (5-30) | 20 (5-37.5) | 30 (15-70) | 15 (5-30) |
| Extranodal extension | p=0.870 | p=0.872 | p=0.465 | p=0.417 |
| Yes | 35 (2.5-47.5) | 25 (15-35) | 22.5 (10-50) | 27.5 (12.5-42.5) |
| No | 30 (5-50) | 20 (10-57.5) | 32.5 (17.5-72.5) | 25 (10-40) |
| Lymphatic invasion | **p=0.031** | p=0.193 | p=0.541 | **p=0.015** |
| Yes | 30 (10-50) | 25 (15-35) | 25 (10-50) | 25 (15-40) |
| No | 15 (0-32.5) | 15 (0-40) | 30 (10-75) | 15 (5-30) |
| Blood vessel invasion | p=0.810 | p=0.424 | p=0.528 | p=0.839 |
| Yes | 20 (0-37.5) | 15 (10-30) | 15 (10-60) | 15 (10-30) |
| No | 20 (5-40) | 25 (10-40) | 27.5 (10-67.5) | 20 (10-35) |
| Infiltrating border | p=0.686 | p=0.630 | **p=0.010** | p=0.973 |
| Yes | 20 (10-35) | 20 (10-30) | 15 (5-25) | 20 (10-32.5) |
| No | 17.5 (0-45) | 22.5 (10-40) | 35 (15-70) | 20 (10-40) |
| Cancer type | p=0.116 | p=0.165 | p=0.102 | p=0.987 |
| Conventional | 15 (0-40) | 20 (10-30) | 25 (10-50) | 20 (10-40) |
| Serrated | 25 (15-55) | 30 (10-55) | 40 (15-80) | 15 (10-35) |
| Mismatch repair (MMR) | p=0.084 | p=0.127 | p=0.428 | p=0.986 |
| Proficient | 20 (2.5-40) | 20 (10-35) | 25 (10-57.5) | 20 (10-35) |
| Deficient | 25 (25-80) | 40 (15-65) | 80 (5-80) | 15 (10-35) |
| *BRAF* mutation | **p=0.033** | p=0.126 | p=0.656 | p=0.233 |
| Yes | 30 (20-80) | 35 (15-65) | 20 (15-80) | 31 (15-35) |
| No | 20 (0-35) | 20 (10-35) | 25 (10-60) | 20 (10-35) |
| *KRAS* mutation | p=0.123 | p=0.751 | p=0.674 | p=0.466 |
| Yes | 25 (12.5-45) | 20 (15-30) | 27.5 (20-50) | 20 (10-35) |
| No | 15 (0-35) | 20 (10-35) | 22.5 (10-65) | 20 (5-35) |

P-values are presented for Mann-Whitney or Kruskall-Wallis tests.
