# Supplementary material for: MUC1 in Colorectal Carcinoma: Association With Prognosis and Putative Anoikis‐Resistant Structures
Source: APMIS. 2025 Dec 2;133(12):e70105. doi: 10.1111/apm.70105 (PMC12673294; doi:10.1111/apm.70105)
Supplement: Supplementary file 3 — Table S3: apm70105‐sup‐0003‐TableS3.docx. [file APM-133-0-s001.docx]

**Supplementary Table 3.** Correlation of MUC1 expression in primary tumors and the corresponding lymph node metastases (n=46). Correlations of proportion of cells showing either membranous or cytoplasmic MUC1 expression between primary tumor tissue and lymph node metastasis were assessed separately for all carcinoma cells, micropapillary (MIPs), cribriform and solid structures by using the Spearman rank correlation test.

| **Primary tumor** | **Lymph node metastases** | | | |
| --- | --- | --- | --- | --- |
| **Membranous MUC1** | All carcinoma cells | MIPs | Cribriform structures | Solid structures |
| All carcinoma cells | 0.343 p=0.024 | 0.353 p=0.037 | 0.342 p=0.031 | 0.356 p=0.031 |
| MIPs | 0.511 p=0.002 | 0.484 p=0.007 | 0.431 p=0.012 | 0.527 p=0.003 |
| Cribriform structures | 0.375 p=0.017 | 0.436 p=0.011 | 0.408 p=0.012 | 0.460 p=0.006 |
| Solid structures | 0.222 p=0.221 | 0.194 p=0.363 | 0.241 p=0.207 | 0.264 p=0.174 |
| **Cytoplasmic MUC1** |  |  |  |  |
| All carcinoma cells | 0.702 p<0.001 | 0.674 p<0.001 | 0.721 p<0.001 | 0.660 p<0.001 |
| MIPs | 0.359 p=0.040 | 0.363 p=0.049 | 0.381 p=0.029 | 0.342 p=0.065 |
| Cribriform structures | 0.663 p<0.001 | 0.674 p<0.001 | 0.696 p<0.001 | 0.626 p<0.001 |
| Solid structures | 0.556 p=0.001 | 0.540 p=0.006 | 0.543 p=0.002 | 0.430 p=0.022 |
